# Supplementary material for: Low-index mesoscopic surface reconstructions of Au surfaces using Bayesian force fields
Source: Nat Commun. 2024 May 6;15:3790. doi: 10.1038/s41467-024-48192-6 (PMC11074279; doi:10.1038/s41467-024-48192-6)
Supplement: Supplementary file 1 — Supplementary Information [file 41467_2024_48192_MOESM1_ESM.pdf]

Supplementary Information for: Low-index mesoscopic surface reconstructions of Au surfaces using Bayesian force fields

Cameron J. Owen†,[1] Yu Xie,[2] Anders Johansson,[2] Lixin Sun‡,[2] and Boris Kozinsky†[2,3]
[1]*Department of Chemistry and Chemical Biology, Harvard University, Cambridge, Massachusetts 02138, United States*
[2]*John A. Paulson School of Engineering and Applied Sciences, Harvard University, Cambridge, Massachusetts 02138, United States*
[3]*Robert Bosch LLC Research and Technology Center, Watertown, Massachusetts 02472, United States*

Supplementary Notes

Suppl. Note 1. MLFF Training and Validation

MLFF Active Learning

As is discussed in the methods section of the main text in more detail, only the Au(111), Au(110), and Au(100) low-index surfaces, as well as bulk supercells were considered in the active-learning simulations performed here. These systems can be visualized in Suppl. Fig. 1, and the active learning simulations are also summarized in Suppl. Table 1. Here, the simulation time (τ_{sim}), temperature (T_{sim}), number of DFT calls (N_{DFT}), number of atoms (N_{atoms}) and total wall-time (τ_{wall}) are recorded. Sums of N_{DFT}, τ_{sim}, and τ_{wall} are provided instead of run-specific information, due to the high number of parallel active learning trajectories considered.

Summarily, only 7 days of parallel CPU wall-time are required to survey 13.2 ns of dynamics by running all active learning trajectories in parallel, where a total of 2965 DFT calls were made. Considering the total number of atoms, systems, and time-steps considered, our active-learning workflow results in an incredible acceleration (on the order of centuries) relative to a pure *ab initio* study of the same systems. Even if all active learning trajectories were run in series, 13.2 ns of dynamics would be observed on the time-scale of 74 days, which is also without any inclusion of FLARE 'warm-starts' using a pretrained GP.

MLFF Validation

In Suppl. Fig. 2 and 3, we provide both validation and parity of the MLFF against DFT labels. In Suppl. Fig. 2, we consider energy versus volume, bulk modulus, elastic constants, surface energies, cohesive energies for a variety of NP sizes, and phonon dispersion for bulk Au. The MLFF yields excellent agreement across all targets, establishing preliminary trust in the MLFF to yield predictions with the same accuracy as DFT across a variety of systems. In Suppl. Fig. 3 we provide a comparison of the MLFF and DFT predictions for energies, forces, and stresses across the entire training set of 2965 frames, where excellent agreement is also observed, as evidenced by the MAEs in each panel.

Suppl. Note 2. Au(111) Reconstruction along the axes of Stoichiometry and Strain

In Suppl. Fig. 4, we provide snapshots of the simulation cells at 0.0% applied mechanical strain to highlight the quick island agglomeration and presence of spinodal decompositions on the Au(111) surface, as a function of adatom coverage. 'Labyrinth' patterns emerge at coverages above 0.4 ML, as was also observed using experimental STM as discussed in the main text. In Suppl. Figures 5 - 15, we provide snapshots of the final simulation cell after 10 ns of production time for each of the combinations considered for strain and surface stoichiometry. Clear differences in the extent of surface reconstruction as a function of both stimuli can be observed.

†Corresponding authors
C.J.O., E-mail: cowen@g.harvard.edu
B.K., E-mail: bkoz@seas.harvard.edu
‡Present address: Microsoft Research, Cambridge, UK

Supplementary Table

System	Temp. (K)	$\sum \tau_{\mathrm{sim}}$ (ns)	$\sum \tau_{\mathrm{wall}}$ (hr)	$\sum N_{\mathrm{DFT}}$	N_{atoms}	N_{runs}
bulk	250-1500	1.22	107.5	252	125	5
Au(111)	250-1500	1.91	455.6	606	96	9
Au(100)	250-1500	1.83	418.7	678	96	9
Au(110)	250-1500	3.73	588.2	1008	96	11
Au_{55}	400-800	2.00	42.4	274	55	6
Au_{147}	500-700	1.40	17.5	41	147	2
Au_{309}	500-800	1.11	145.2	106	309	4

Supplementary Table 1. Summary of the FLARE active-learning trajectories for each of the Au systems.

Supplementary Figures

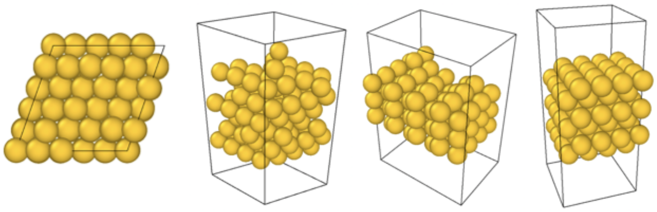

Supplementary Figure 1. Summary of all systems considered for active learning. From left to right: bulk Au, Au(111) including an adatom and vacancy pair, Au(110), and Au(100).

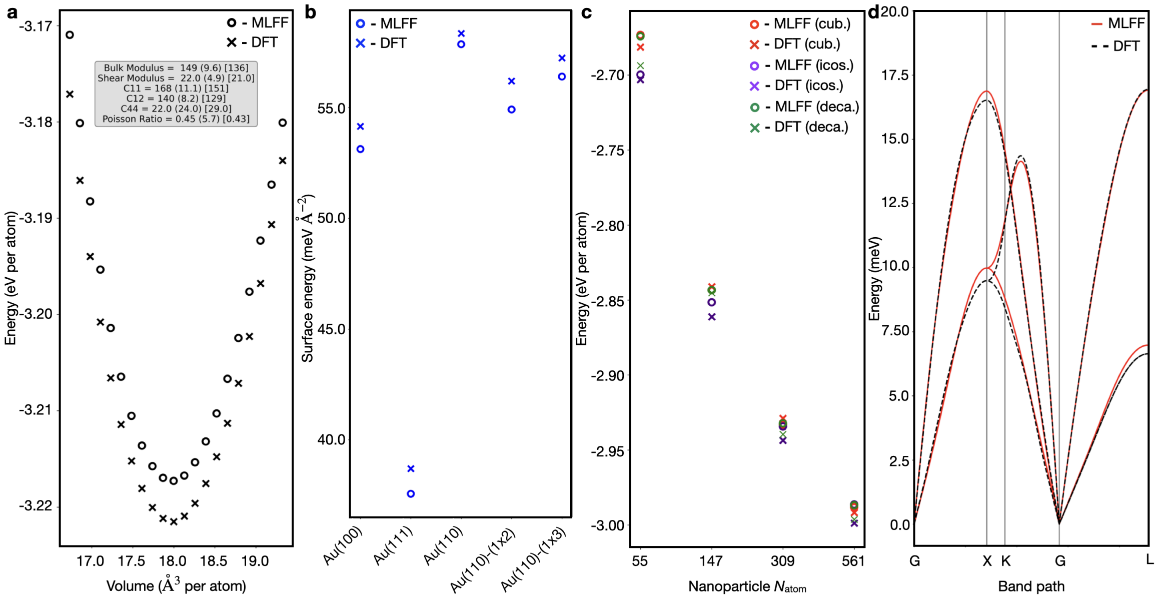

Supplementary Figure 2. **a** Energy versus volume comparison between DFT (crosses) and the MLFF (open circles), and performance on bulk targets (percent error given in parentheses relative to DFT, with DFT value given in brackets). **b** Surface energy (eV per Å) comparison between the MLFF (open circles) and DFT (crosses). **c** Total energy-per-atom comparison between the MLFF (open circles) and DFT (crosses) for NPs of different shapes and sizes. **d** Phonon dispersion comparison between the MLFF (red) and DFT (black).

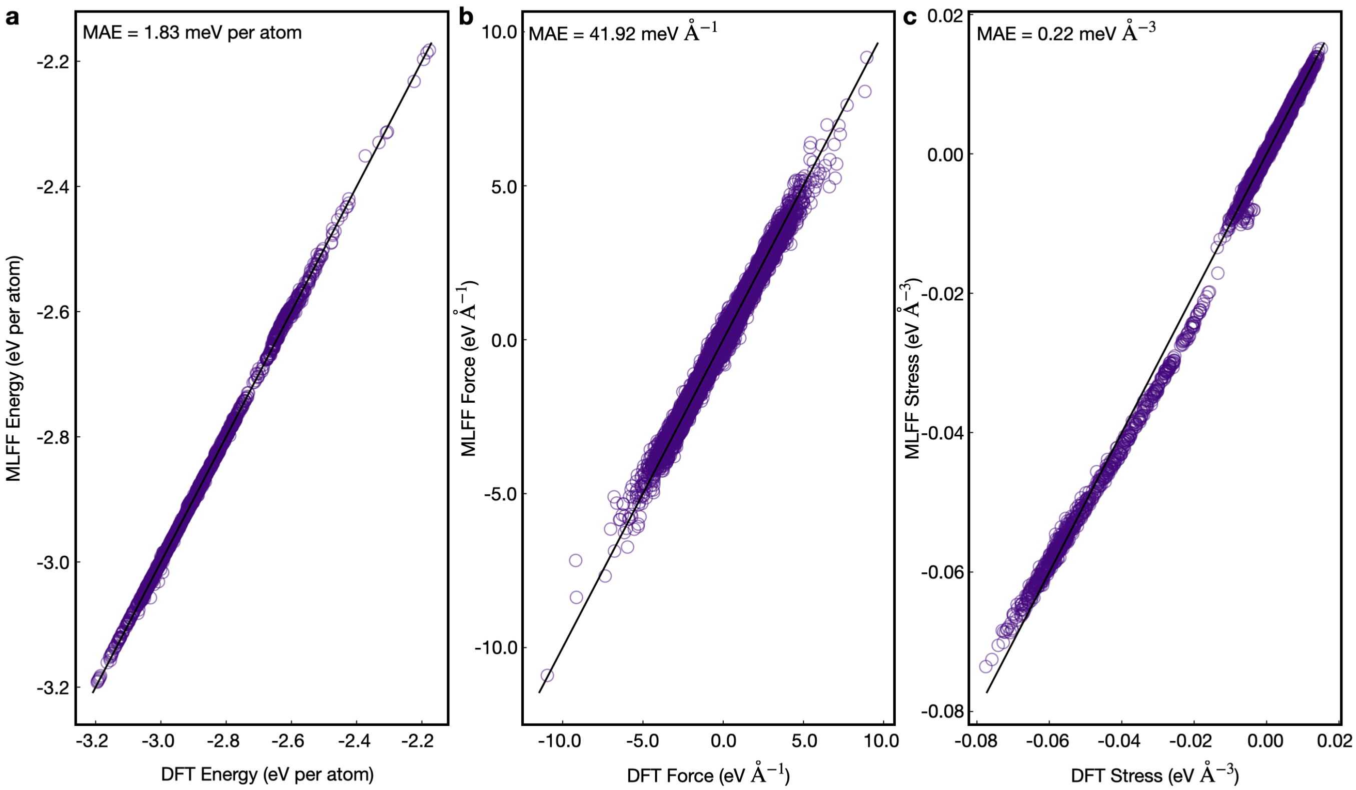

Supplementary Figure 3. Parity results for the MLFF against DFT on the entire training set. **a** energy, **b** force, and **c** stress labels and their respective errors.

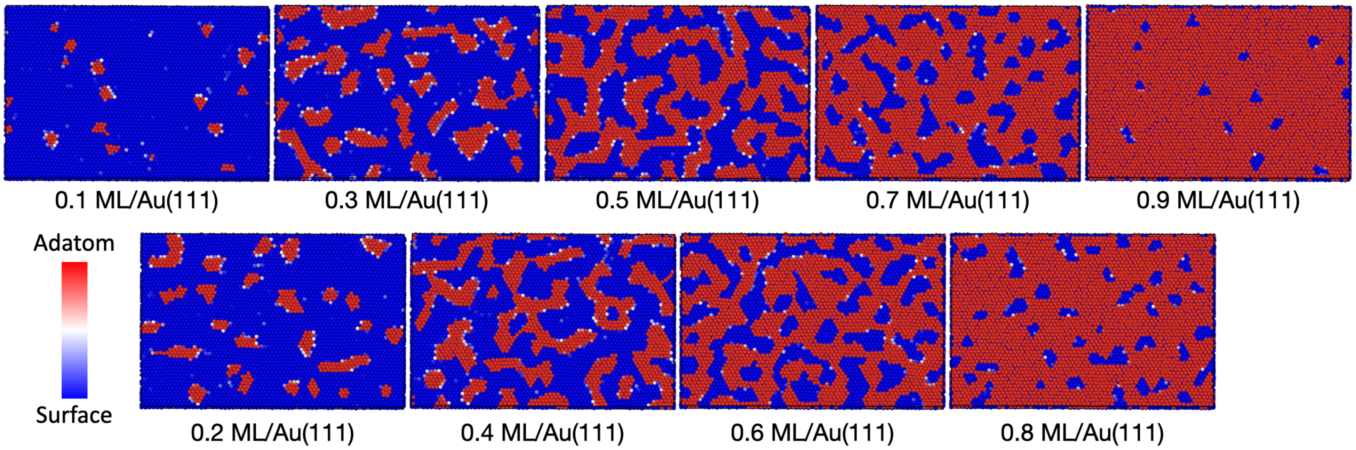

Supplementary Figure 4. Snapshots of the final simulation cells (taken at 10 ns) for Au(111) surfaces with varying levels of adatom coverage evolved at 300 K without the application of mechanical strain. The atoms are colored by their heights in the surface-normal direction to allow for identification of the spinodal decompositions that appear and persist throughout the simulations.

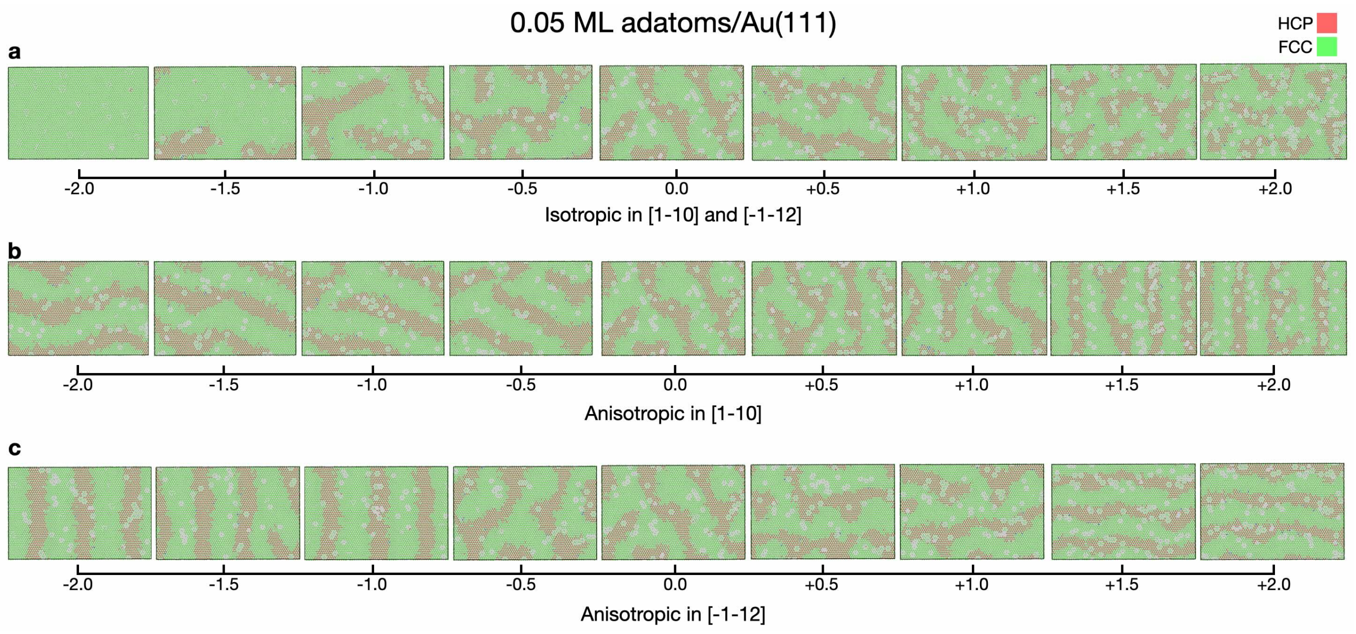

Supplementary Figure 5. Coupled results of strain applied to the 0.05 ML adatoms/Au(111) system. Atoms are colored by their atomic environment using the Polyhedral Template Matching method in Ovito and applied strain values are provided (in values of %). **a** Isotropic strain applied along both the $[1\hat{1}0]$ and $[\hat{1}\hat{1}2]$ lattice vectors. **b** Anisotropic strain applied along the $[1\hat{1}0]$ direction. **c** Anisotropic strain applied along the $[\hat{1}\hat{1}2]$ direction.

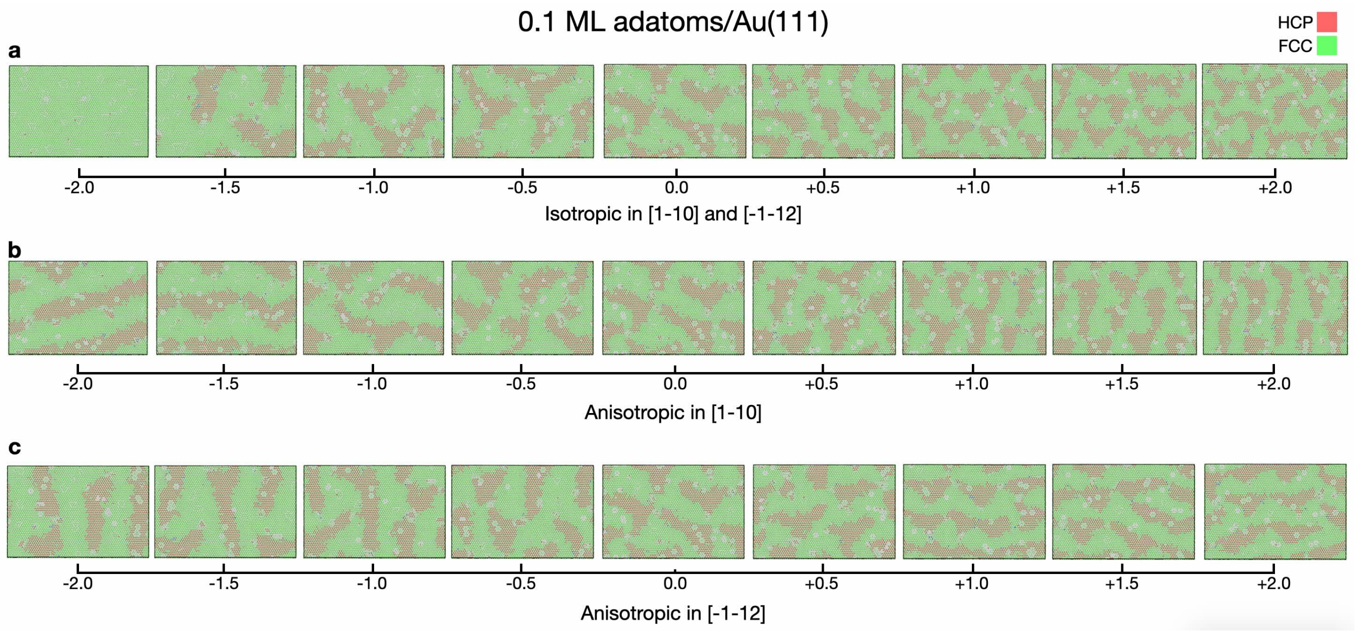

Supplementary Figure 6. Coupled results of strain applied to the 0.1 ML adatoms/Au(111) system. Atoms are colored by their atomic environment using the Polyhedral Template Matching method in Ovito and applied strain values are provided (in values of %). **a** Isotropic strain applied along both the $[1\hat{1}0]$ and $[\hat{1}\hat{1}2]$ lattice vectors. **b** Anisotropic strain applied along the $[1\hat{1}0]$ direction. **c** Anisotropic strain applied along the $[\hat{1}\hat{1}2]$ direction.

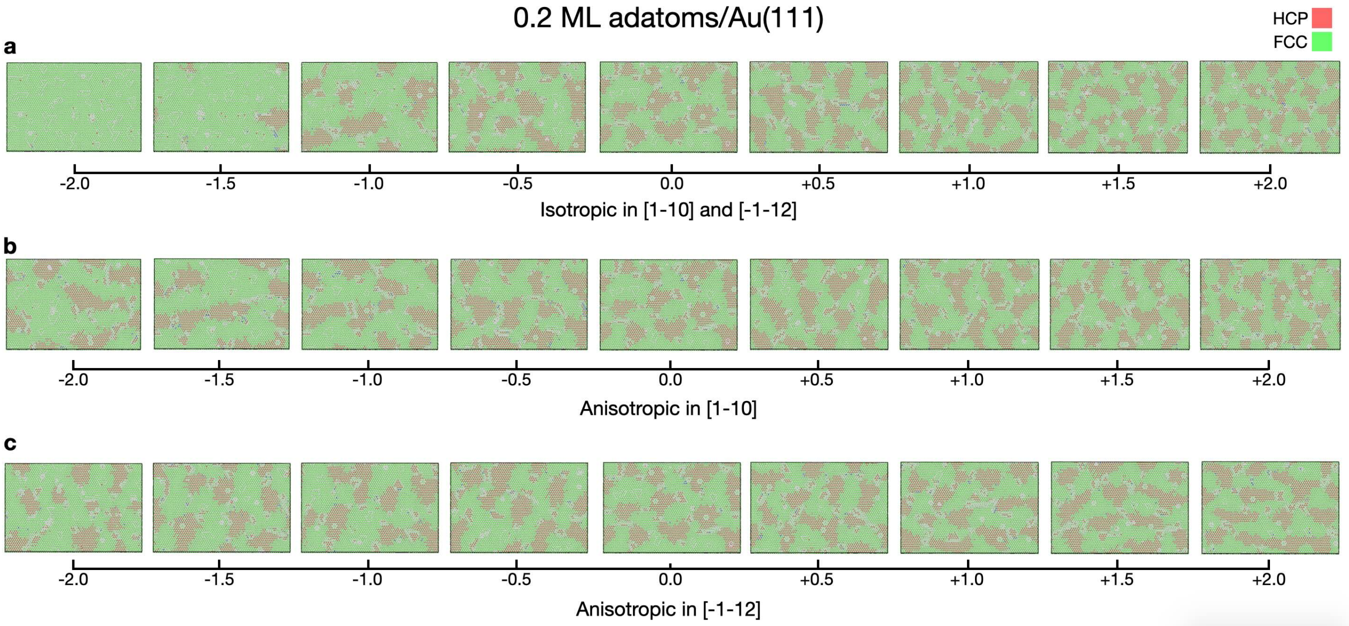

Supplementary Figure 7. Coupled results of strain applied to the 0.2 ML adatoms/Au(111) system. Atoms are colored by their atomic environment using the Polyhedral Template Matching method in Ovito and applied strain values are provided (in values of %). **a** Isotropic strain applied along both the $[1\hat{1}0]$ and $[\hat{1}\hat{1}2]$ lattice vectors. **b** Anisotropic strain applied along the $[1\hat{1}0]$ direction. **c** Anisotropic strain applied along the $[\hat{1}\hat{1}2]$ direction.

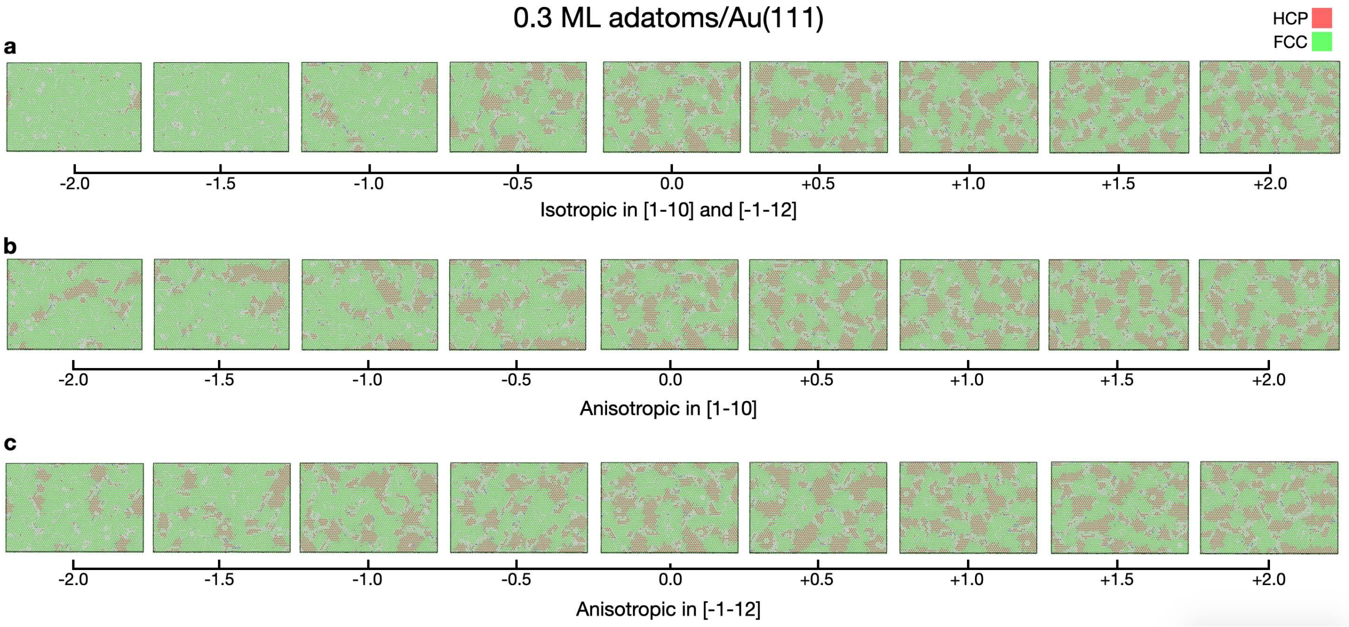

Supplementary Figure 8. Coupled results of strain applied to the 0.3 ML adatoms/Au(111) system. Atoms are colored by their atomic environment using the Polyhedral Template Matching method in Ovito and applied strain values are provided (in values of %). **a** Isotropic strain applied along both the $[1\hat{1}0]$ and $[\hat{1}\hat{1}2]$ lattice vectors. **b** Anisotropic strain applied along the $[1\hat{1}0]$ direction. **c** Anisotropic strain applied along the $[\hat{1}\hat{1}2]$ direction.

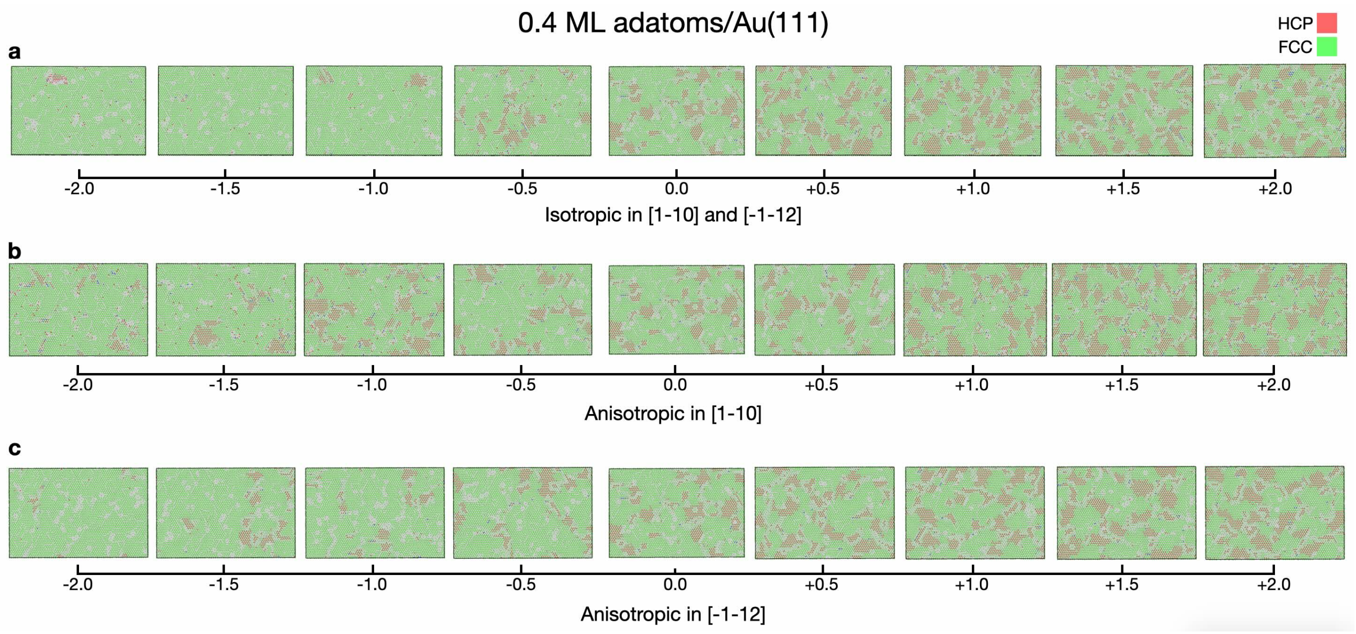

Supplementary Figure 9. Coupled results of strain applied to the 0.4 ML adatoms/Au(111) system. Atoms are colored by their atomic environment using the Polyhedral Template Matching method in Ovito and applied strain values are provided (in values of %). **a** Isotropic strain applied along both the $[1\hat{1}0]$ and $[\hat{1}\hat{1}2]$ lattice vectors. **b** Anisotropic strain applied along the $[1\hat{1}0]$ direction. **c** Anisotropic strain applied along the $[\hat{1}\hat{1}2]$ direction.

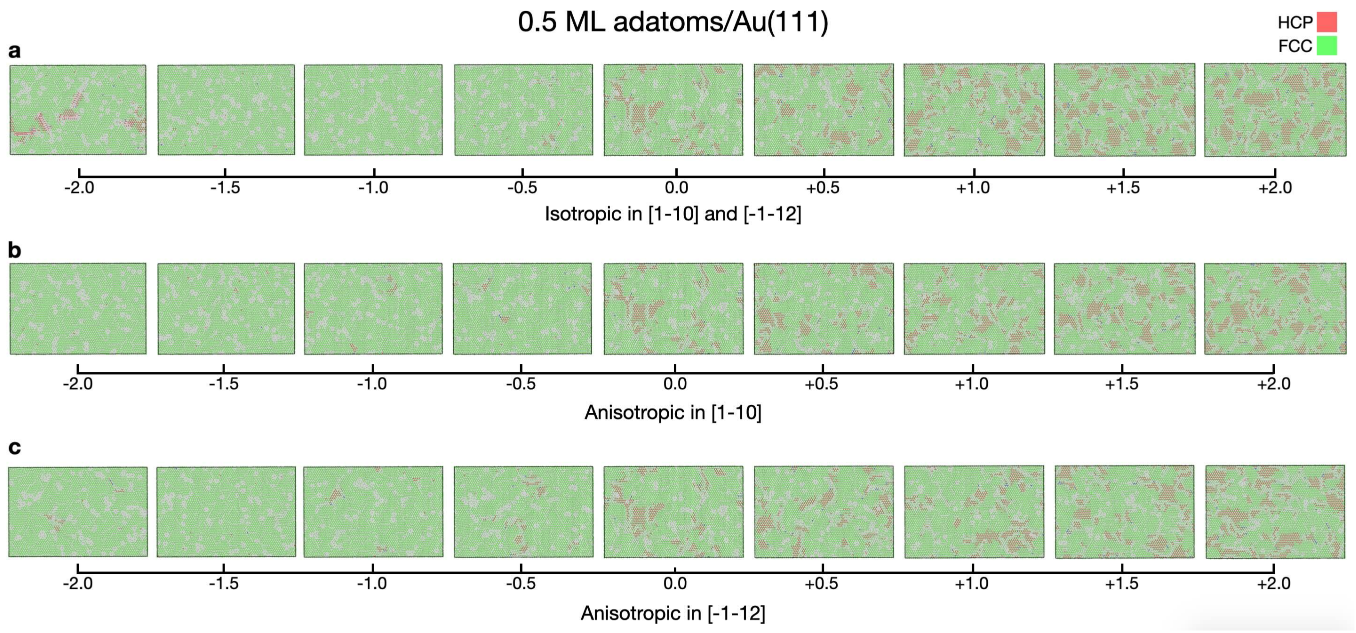

Supplementary Figure 10. Coupled results of strain applied to the 0.5 ML adatoms/Au(111) system. Atoms are colored by their atomic environment using the Polyhedral Template Matching method in Ovito and applied strain values are provided (in values of %). **a** Isotropic strain applied along both the $[1\hat{1}0]$ and $[\hat{1}\hat{1}2]$ lattice vectors. **b** Anisotropic strain applied along the $[1\hat{1}0]$ direction. **c** Anisotropic strain applied along the $[\hat{1}\hat{1}2]$ direction.

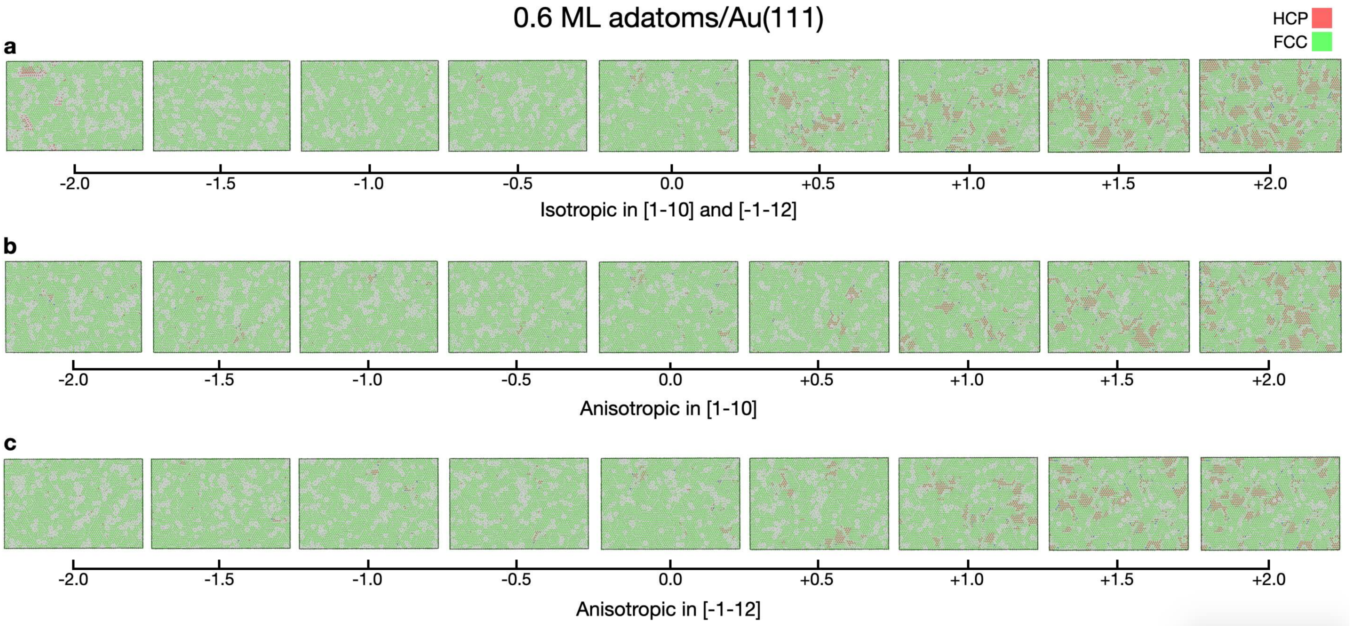

Supplementary Figure 11. Coupled results of strain applied to the 0.6 ML adatoms/Au(111) system. Atoms are colored by their atomic environment using the Polyhedral Template Matching method in Ovito and applied strain values are provided (in values of %). **a** Isotropic strain applied along both the $[1\hat{1}0]$ and $[\hat{1}\hat{1}2]$ lattice vectors. **b** Anisotropic strain applied along the $[1\hat{1}0]$ direction. **c** Anisotropic strain applied along the $[\hat{1}\hat{1}2]$ direction.

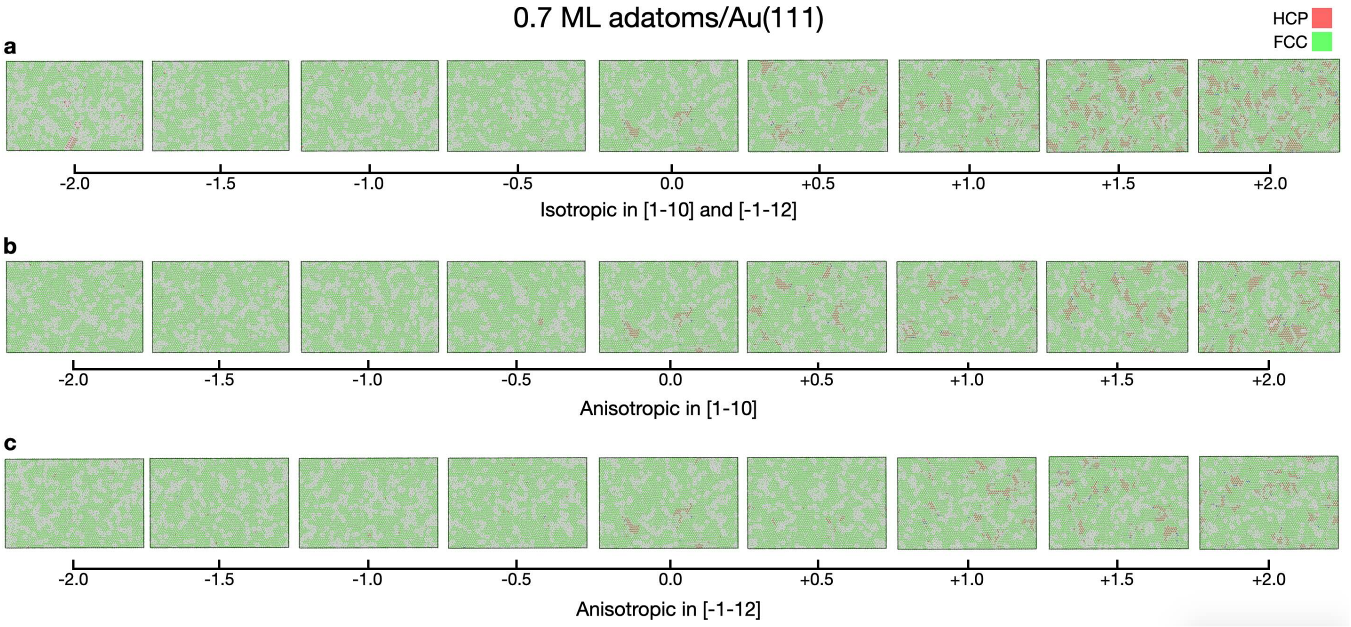

Supplementary Figure 12. Coupled results of strain applied to the 0.7 ML adatoms/Au(111) system. Atoms are colored by their atomic environment using the Polyhedral Template Matching method in Ovito and applied strain values are provided (in values of %). **a** Isotropic strain applied along both the $[1\hat{1}0]$ and $[\hat{1}\hat{1}2]$ lattice vectors. **b** Anisotropic strain applied along the $[1\hat{1}0]$ direction. **c** Anisotropic strain applied along the $[\hat{1}\hat{1}2]$ direction.

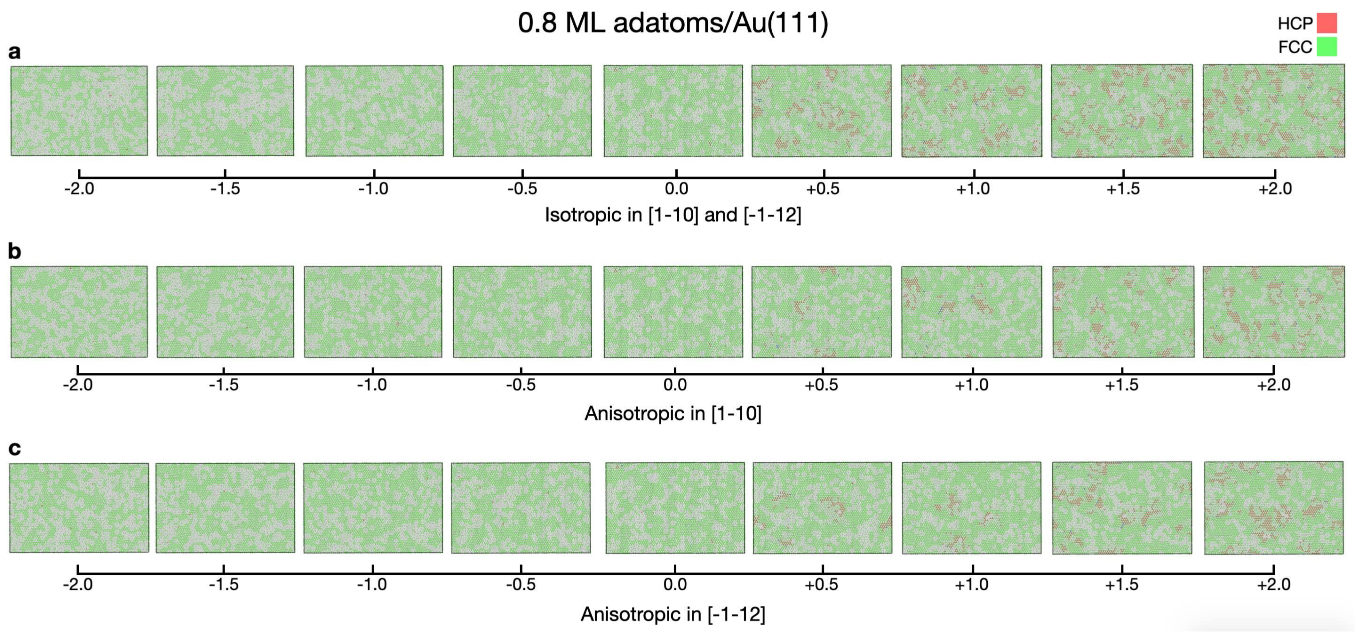

Supplementary Figure 13. Coupled results of strain applied to the 0.8 ML adatoms/Au(111) system. Atoms are colored by their atomic environment using the Polyhedral Template Matching method in Ovito and applied strain values are provided (in values of %). **a** Isotropic strain applied along both the $[1\hat{1}0]$ and $[\hat{1}\hat{1}2]$ lattice vectors. **b** Anisotropic strain applied along the $[1\hat{1}0]$ direction. **c** Anisotropic strain applied along the $[\hat{1}\hat{1}2]$ direction.

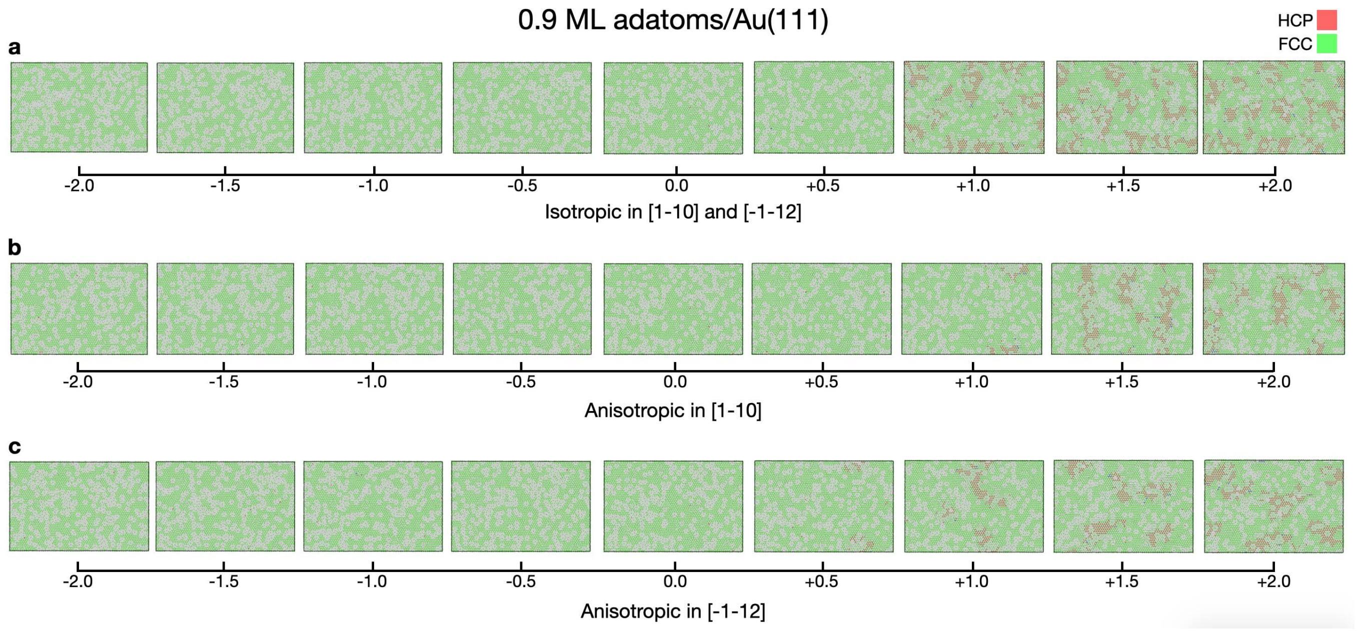

Supplementary Figure 14. Coupled results of strain applied to the 0.9 ML adatoms/Au(111) system. Atoms are colored by their atomic environment using the Polyhedral Template Matching method in Ovito and applied strain values are provided (in values of %). **a** Isotropic strain applied along both the $[1\hat{1}0]$ and $[\hat{1}\hat{1}2]$ lattice vectors. **b** Anisotropic strain applied along the $[1\hat{1}0]$ direction. **c** Anisotropic strain applied along the $[\hat{1}\hat{1}2]$ direction.

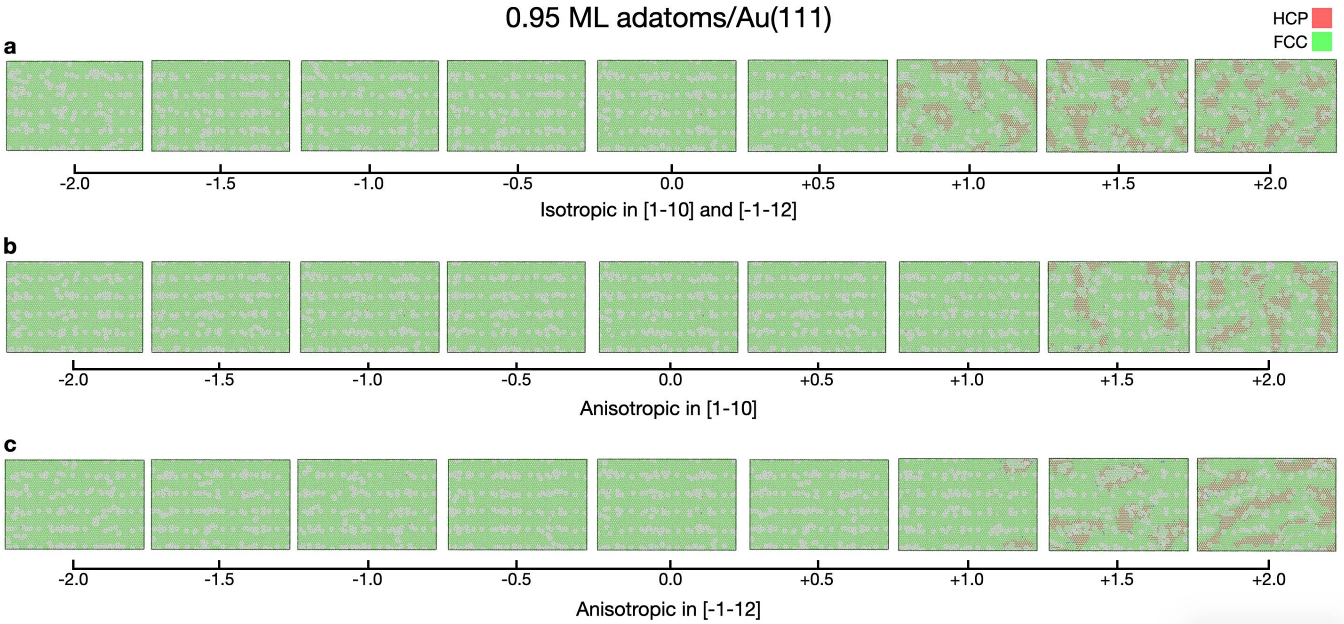

Supplementary Figure 15. Coupled results of strain applied to the 0.95 ML adatoms/Au(111) system. Atoms are colored by their atomic environment using the Polyhedral Template Matching method in Ovito and applied strain values are provided (in values of %). **a** Isotropic strain applied along both the $[1\hat{1}0]$ and $[\hat{1}\hat{1}2]$ lattice vectors. **b** Anisotropic strain applied along the $[1\hat{1}0]$ direction. **c** Anisotropic strain applied along the $[\hat{1}\hat{1}2]$ direction.
